# Autism Awareness Month and World Autism Day — April 2013

**Published:** 2013-03-29

April is Autism Awareness Month, and April 2 is World Autism Day. Nearly one in 88 children has been identified with an autism spectrum disorder (ASD), according to estimates from CDC’s Autism and Developmental Disabilities Monitoring (ADDM) Network (1). ASDs are a group of developmental disabilities that can result in major social, communication, and behavioral challenges. Onset of symptoms usually occurs between a child’s first and third birthdays (1). Early identification and intervention can help a child access services and learn new skills; however, most children are not identified until after they reach age 4 years (1).

ADDM Network surveillance data serve as a guide for CDC’s own autism research as well as the research of other scientists throughout the United States. To identify the causes of ASDs, the scientific community first needs to better understand the factors that put children at risk for ASDs. CDC currently is conducting the Study to Explore Early Development to help identify these risk factors (2).

CDC’s “Learn the Signs, Act Early” program (http://www.cdc.gov/actearly) has tools to help parents and early childhood-care and education providers track children’s developmental milestones and provides information about what to do if there is a concern. This program also offers resources for health-care providers, including the Autism Case Training course, which is available online for individual continuing education credit and as a classroom-based curriculum for pediatric residency programs. Additional information is available at http://www.cdc.gov/autism.
